# Effects of Balsa Fish Skin Gelatin, *Lentinula edodes* Mushrooms, Soy Protein Isolate, and Starch on the Sensory Quality and Characterization of Physicochemical and Antioxidant Properties of New Sausage

**DOI:** 10.3390/foods13030465

**Published:** 2024-02-01

**Authors:** Liyan Wang, Jiacheng Yin, Kang Wan, Hongyue Guo, Guochuan Jiang

**Affiliations:** College of Food Science and Engineering, Jilin Agricultural University, 2888 Xincheng Street, Changchun 130118, China; wangliyan@jlau.edu.cn (L.W.); 20231092@mails.jlau.edu.cn (J.Y.); wankang@jlau.edu.cn (K.W.); guohongyue@jlau.edu.cn (H.G.)

**Keywords:** balsa fish skin gelatin, sausages, *Lentinula edodes*, antioxidant capacity

## Abstract

Sausages are loved by people for their unique texture, satisfying chewiness, and pleasant flavor. However, in the production of sausages, red meat and a large amount of fat are mainly used, and long-term consumption will increase the risk of diseases such as obesity, heart disease, hypertension, and cancer. Our previous studies have shown that the intake of red meat and fat can be reduced through the replacement of lean meat and fat in sausages by *Lentinula edodes* and *Pleaurotus eryngii* mushrooms, but this will lead to the deterioration of the gel of sausage products and seriously affect the sensory quality of sausages. In this study, the response surface method was used to optimize the amount of balsa fish skin gelatin, soy protein isolate, and starch added to, and the proportion of *Lentinula edodes* mushrooms replacing lean meat in, the new sausage, with *Pleaurotus eryngii* mushrooms replacing fat. The results show that Lentinula edodes mushrooms replaced 36.1% of the lean meat, and the addition of 0.96% balsa fish skin gelatin, 10.61% starch, and 9.94% soy protein isolate resulted in the highest sensory score and the sensory quality being the closest to that of traditional sausages. Compared with the control group, this novel sausage exhibits characteristics such as lower fat and saturated fatty acid content, reduced energy levels, and higher levels of amino acids (aspartic acid, glutamic acid, cysteine, methionine, and proline) and polyunsaturated fatty acids. The total phenolic content of the novel sausage is 12.52 times higher than that of the control. In comparison with the control group, the novel sausage demonstrates a 65.58% increase in DPPH radical scavenging activity and a 3.88-fold improvement in ABTS+ radical scavenging activity. These findings highlight the outstanding antioxidant performance of the novel sausage. This study provides new ideas for improving the sensory quality of new sausages, promoting the healthy development of the sausage industry, and promoting the high-value utilization of edible mushrooms.

## 1. Introduction

Sausage products, known for the convenience of their consumption and their delectable taste, enjoy popularity among consumers [1]. However, concerns arise as the intake of red meat has been linked to an increased risk of colorectal cancer by the International Agency for Research on Cancer [2]. Additionally, increased fat consumption is associated with elevated risks of obesity, heart disease, hypertension, and various cancers [3], posing challenges to the progression of the meat industry [4]. Despite the nutritional value of red meat and fat, there is a pressing need to curtail their intake. Hence, as indicated by advancements in meat science, the development of sausages with reduced red meat and fat content holds promising market prospects, offering consumers a healthier alternative compared with traditional sausages.

Mushrooms serve as an excellent source of protein and dietary fiber in nutrition. Due to their nutritional and medicinal value, they have been consumed for several centuries [5,6,7]. *Lentinula edodes* (*L. edodes*) and *Pleurotus eryngii* (*P. eryngii*) belong to the mushroom family and are regarded as edible and medicinal. Both mushrooms are rich plant sources of protein, contributing to the maintenance of physiological functions and tissue repair [2,3]. The abundance of amino acids, including glutamic acid and serine, provides high nutritional value. The dietary fiber and vitamins present in mushrooms play a crucial role in promoting human health [8]. Numerous studies indicate that *L. edodes* mushrooms not only promote digestion, regulate bowel movements, protect the gastrointestinal tract, reduce the number of fat cells, prevent obesity, and mitigate metabolic diseases but also enhance the body’s immune function, slow the spread of tumor cells, and exhibit good resistance to certain viruses. *L. edodes* polysaccharides, which have minimal toxic side effects compared with other anti-tumor drugs, are recognized as some of the most potent immune enhancers. Additionally, research has found that *L. edodes* mushrooms, through purines and polysaccharides, promote cholesterol metabolism, thereby lowering its serum levels and achieving anti-thrombotic effects [9,10,11,12]. Furthermore, the protein in *L. edodes* mushrooms rivals that in meat and eggs, yet their fat and cholesterol contents are significantly lower than those in meat [2], providing a meat-like texture when consumed. Therefore, mushrooms demonstrate potential as substitutes for meat. Our early studies indicated that *L. edodes* and *P. eryngii* could be used as substitutes for lean meat and fat, respectively, to produce sausages with low lean meat or low fat content [2,3]. However, there have been no reports on the use of *L. edodes* mushrooms as a substitute for lean meat and *P. eryngii* mushrooms as a substitute for fat in a new type of healthy sausage. According to our early research results, the increase in the proportion of *L. edodes* mushrooms replacing lean meat has led to a deterioration in the gelatinicity of sausage products, which has seriously affected the sensory quality of the sausages. This is an important issue that urgently needs to be addressed in the industrial development of new healthy sausages.

When meat is replaced by other ingredients, the texture and sensory properties of sausage products are affected. In order to overcome the loss of quality in meat or meatless sausages, various plant proteins (such as legumes and grain proteins) and starch can be used to improve the sensory quality of the product [13,14]. The functional characteristics of soybean protein isolate (SPI), such as emulsification, water absorption, and oil absorption, make it a unique meat substitute [15,16]. SPI has gelatinous and viscous properties, which can effectively improve the texture of meat products, increase the elasticity of products, and improve the quality of products [17]. At the same time, SPI can create a sticky gel matrix to hold moisture and fat, thus making the emulsion stable. SPI helps to improve the hardness of the batter system by supplementing myosin and actin as a fat coating agent, preventing fat separation during cooking [18]. In addition, soy proteins and starch establish a strong network that can physically capture water, thereby increasing the water holding capacity. Starch can simulate the sensory properties of fat and can also combine with the moisture in meat products to maintain the juiciness and tenderness of sausages. Research has shown that the application of starch in steamed sausages can significantly improve their elasticity and cohesiveness. From this, it can be seen that adding SPI and starch to low-meat and low-fat sausages plays an important role in improving the tissue structure, water retention, and sensory quality of the product. It can achieve more ideal results for low-meat and low-fat meat products.

*Pangasius bocourti*, commonly known as balsa fish, is a fish species of significant economic importance that is mainly found in the Mekong River Delta [19]. The by-product of balsa fish processing, namely balsa fish skin, contains collagen at approximately 70% of its dry weight [20]. Extensive research has confirmed that balsa fish skin gelatin (BFSG) is a potential source of angiotensin-converting enzyme (ACE) inhibitory peptides, making it a promising and valuable functional food [21]. Studies have indicated that the application of gelatin to hamburger patties can improve their textural properties and enhance their overall palatability [22]. Compared with mammalian gelatin, balsa fish skin gelatin exhibits a higher viscosity and lower gelation and melting temperatures. Therefore, balsa fish skin gelatin (BFSG) has promising prospects in meat product applications. Research on the incorporation of balsa fish skin gelatin in sausage processing is crucial for the value-added utilization of balsa fish by-products and the enhancement of the processing quality of healthy sausages. Modern medical research suggests that various diseases, such as aging, tumors, and coronary heart disease, are associated with oxidative damage to membrane lipids, which is caused by excessive free radicals [23]. Therefore, the daily intake of sausages with high antioxidant properties will play an important role in maintaining people’s physical health.

This study optimized the proportion of *L. edodes* mushrooms replacing lean pork and the amount of Balsa fish skin gelatin, soy protein isolate, and starch added through the response surface methodology to improve the sensory quality of a new type of sausage. Subsequently, the physicochemical and antioxidant properties of the new sausage were compared with those of sausages that did not replace the lean meat and fat. This study obtained a healthy sausage formula with antioxidant properties that improves the sensory quality of new sausages and provides new insights for the healthy development of the sausage industry and the high-value utilization of edible mushrooms.

## 2. Materials and Methods

### 2.1. Materials

The pork lean meat (pork hind leg; protein, 21.13%; fat, 6.25%; moisture, 69.38%; ash, 1.05%), pork back-fat, *P. eryngii*, and corn oil were purchased from a local market. The dry *L. edodes* mushrooms (protein, 21.09%; fat, 1.22%; moisture, 8.31%; ash, 4.91%; total dietary fiber, 31.61%) were supplied by Hubei Yuguo Mushroom Co., Ltd. (Suizhou, China). All additives, chemical reagents (analytical reagents), and BFSG (food additive grade) were obtained from Changchun Yibo Biotechnology Co., Ltd. (Changchun, China). The dry starch (DS) and SPI were purchased from Linyi Mountain Pine Biological products Co., Ltd. (Linyi, China).

### 2.2. Experimental Design

The formulation of the novel sausage was optimized by using the response surface methodology (RSM), which was applied to investigate the simultaneous effects of four formulation variables (*L. edodes*, x1; BFSG, x2; DS, x3; SPI, x4) and conducted in a 27-run experiment to determine the optimal levels (Table 1). Each experiment was repeated three times and the average score of the sensory evaluation was used as the response value (Y). The experiments were based on a Box–Behnken design. Each factor or variable was provided for on three levels. Using sensory evaluation as an indicator, we validated the formula optimized by the response surface methodology to further determine the optimal formula.

### 2.3. Formulations and Processing

We prepared a total of 28 different formulations, comprising a control group and 27 experimental groups. The control group was prepared with 80% pork lean meat and 20% pork back fat. After washing, the lean meat was cut into 2–3 cm thick pieces, and the fat was cut into 1.5 cm square blocks. The amount of additive used for sample preparation was based on the total percentage of pork lean meat and pork back fat. The lean meat and fat were thoroughly mixed with seasoning (2.5% salt and 0.5% vitamin C), compacted, covered with plastic wrap, and marinated at 4 °C for 24 h. Simultaneously, the casings were soaked for 24 h. Subsequently, the lean meat, 1% sugar, 1% monosodium glutamate, 0.12% sodium pyrophosphate, 0.08% sodium tripolyphosphate, spices, carrageenan (dissolved in water), 2.84% isolated soy protein (dissolved in water), 4.26% starch, red koji rice extract (dissolved in water), water (dissolved), and fat were sequentially placed into a mixer (Busch, Marburg, Germany) and homogenized for 140 s in each batch. The mixture was then stuffed into casings, tied every 12 cm, surface-washed with water, and hung on meat racks. The final steps included baking at 68 °C for 30 min, steaming at 80 °C for 50 min, and smoking at 50 °C for 150 min, all carried out by a smoking chamber (YX-1/1-E-type smoking chamber, produced by Kesi Machinery Equipment Co., Ltd., Jiaxing, China).

The preparation of sausage samples for the experimental groups Involved different ratios of marinated lean meat, processed *P. eryngii* (*P. eryngii* mushrooms cut into small squares with a side length of about 1.5 cm, then fried in corn oil at 160 °C for 2 min), and substitutes for all fat. The processed *P. eryngii* mushrooms were added in accordance with the ratios in Table 2. Other steps were consistent with those of the control group. We added processed *L. edodes* mushrooms according to the ratios in the response surface experiment design. We soaked dried *L. edodes* mushrooms in water for 24 h, separated the cap and stem, treated the stem in a sodium carbonate solution, and heated both parts in peanut oil at 70 °C for 30 min to eliminate the mushroom flavor. After scraping off the black substance, modified *L. edodes* mushrooms were obtained (a red pigment from the peanuts was added for coloring). DS, SPI, and BFSG (BFSG mixed with water in a 1:4 ratio for 12 h and melted in warm water at 60 °C for 8 h), along with other additives, were placed into a mixer (Busch, Marburg, Germany) for homogenization. The subsequent steps taken were the same as those for the control group.

### 2.4. Sensory Evaluation

In accordance with our prior investigation [2], sensory evaluations of the samples were conducted by a panel of nine trained members. In order to protect the rights of participants, we signed informed consent forms with nine sensory evaluation volunteers. The ingredients for new sausages were food ingredients and food additives allowed in food. The research does not involve human ethical issues. The panel, consisting of 4 males and 5 females with an average age of 31 (ranging from 23 to 57), was drawn from the College of Food Science and Engineering at Jilin Agricultural University. These panelists possessed a sound familiarity with meat products and underwent a rigorous training regimen, encompassing a total of 6 sessions, each lasting 1.5 h, prior to the sensory evaluation.

Throughout the training period, the panelists consistently assessed and examined the overall acceptability of diverse samples. Training involved the use of 5 commercial samples, with discussions continuing until a consensus on their overall acceptability was reached. For the formal evaluation, the 27 experimental samples were randomly assigned numbers from 1 to 29. Sensory tests were conducted on different days during six separate sessions, with each session featuring the same panelists. Samples 1–5 were evaluated in the first session, samples 6–10 were evaluated in the second session, and so on, concluding with samples 26–27 in the sixth session.

To standardize the evaluation conditions, the samples were reheated to approximately 25 °C using a microwave oven, sliced to 1.5 cm in thickness, and presented on white plates to the panelists. Palate cleansing between samples was facilitated with room-temperature water and unsalted crackers. The overall acceptability of the samples was assessed using a 10-point hedonic scale, ranging from 1 (intensely dislike) to 10 (intensely like).

### 2.5. Proximate Composition, Energy Value, and Color

The water, ash, fat, and protein contents of samples were detected according to AOAC (2005). The energy value was calculated on the basis of 9 kcal/g for fat, 4 kcal/g for protein, and 4 kcal/g for carbohydrates [24]. The color of samples was measured according to our previous studies [2] and the external color and internal color of sausages were examined by using a HunterLab ColorFlex (illuminant, D65; standard observer, 10°; Xinlian Creation Electronic Co., Ltd., Shanghai, China). The aperture of the meter was 14 mm. The color values of L* (luminosity), a* (negative—green; positive—red), and b* (negative—blue; positive—yellow) were measured. A standard CX 2064 plate was used as a standard (L* = 52.08, a* = −26.39, b* = 14.88). The total color difference (ΔE*) was calculated as follows:

(1) $$∆E=\left(∆a^{2}+∆b^{2}+∆L^{2}\right)1/2$$

where Δa = a* − a*sample, Δb = b* − b*sample, ΔL = L* − L*sample.

### 2.6. Amino Acid Profile

The amino acid content was determined according to Jo et al. [25]. The sample (1 g) was hydrolyzed with 6 mol/L hydrochloric acid (10 mL) for 24 h at 110 °C, filtered, concentrated at 55 °C, mixed with vortex (10 μL), borate buffer solution (70 μL), and OPA derivatizing agent (20 μL) for 1 min, and then placed in an oven at 55 °C for 10 min. The amino acid content was analyzed by using Acquity h-class Ultra Performance Liquid Chromatography (e2695, Waters, Milford, MA, USA).

### 2.7. Fatty Acid Profile

The extraction of lipids from the samples was performed using the method described by Folch, Lees, and Stanley [26], and the fatty acid methyl esters (FAMEs) were prepared according to AOCS Ce 2–66 [27]. FAMEs were analyzed using an Agilent 7890 gas chromatography instrument coupled with an Agilent MS-5975 (Agilent Technologies). An HP-5MS capillary column with dimensions of 60 m × 0.25 mm id × 0.25 μm film thickness was applied for the separation of FAMEs. The temperature profile of the oven was 120 °C for 1 min followed by an increase at 6 °C/min to 170 °C for 0 min, an increase at 2.5 °C/min to 215 °C for 12 min, an increase at 4 °C each minute to 230 °C for 10 min, and finally an increase at 10 °C/min to 280 °C for 15 min. The injector temperature, ion source, quadrupole, and GC/MS interface were 260 °C, 200 °C, 150 °C, and 280 °C, respectively. The conditions applied for gas chromatography were nitrogen as the carrier gas at a flow of 1.5 mL/min and a split ratio of 1:20. The mass spectrometry was performed in the electron impact (EI) mode at 70 eV in the scan range of 40–550 *m*/*z*. The major fatty acids were identified by comparing their retention times with those of pure standards (GLC NESTLE 37 MIX, ANPEL Laboratory Technologies Inc., Beijing, China). The area under the peaks was calculated with the software attached to the instrument. Then, each fatty acid was calculated as mg/100 g of the sample.

### 2.8. Volatile Compound Analysis

The volatile compounds in samples were analyzed by headspace solid-phase microextraction and gas chromatography–mass spectrometry (Agilent 5975-6890N, Palo Alto, CA, USA) as described by Liu et al. [28]. The sample (4 g) was placed in a 15 mL vial, sealed with a PTFE/silicone septum, and heated at 45 °C for 30 min. Then, the SPME fiber was exposed to the headspace at 50 °C for 30 min. The adsorbed compounds were desorbed in splitless mode at 250 °C at the injection port. Molecules were separated using an HP-INNOWax Polyethylene Glycol column (30 m × 0.25 mm × 0.25 μm, Agilent 19091N-133, Palo Alto, CA, USA) with a temperature program of 37 °C for 30 min, an increase to 280 °C at a rate of 4 °C/min, and then 280 °C for 30 min. The volatile compounds detected were identified by comparison with the mass spectra in the NIST’ 08 database and linear retention index (LRI) values of authentic standards or the literature. Quantification was performed by the total extracted area and each of the results is presented as the mean of three different replications.

### 2.9. Total Phenolic Content

The total phenolic content (TPC) was determined spectrophotometrically using the method reported by Półtorak et al. [29]. The standard curve was plotted with an aqueous gallic acid solution at concentrations of 0–16 μg/mL. The sample (2.5 g) was homogenized with 7.5 mL of ethanol, and then 0.7 mL of the extract was mixed with 6 mL of water and 0.5 mL of forintanol reagent for 3 min. Further, 1.5 mL of 20 wt% sodium carbonate solution was added, and water was added to fix the volume to 10 mL. The solution was then incubated in water at 40 °C for 30 min. The absorbance of the mixture at a wavelength of 765 nm was measured. The results represent an average of three replicates in gallic acid equivalents (mg/g) of the sample.

### 2.10. DPPH Radical Scavenging Activity

The scavenging activity on DPPH free radicals was used to determine the antioxidant activity according to the method reported by Półtorak et al. [29]. Briefly, a minced sample (2.5 g) was mixed with 7.5 mL of ethanol (analytical reagent) and homogenized for 2 min using a high-speed blender (Ultraturrax T-25, IKA Labortechnik, Staufen, Germany) (12,000 rpm). Then, the mixture was shaken for 10 min. After centrifugation at 1800 rpm for 10 min, 0.5 mL of the supernatant was mixed with 3.5 mL of 0.1 mM DPPH ethanolic solution and incubated in a dark room for 20 min. The absorbance was measured at 517 nm and the percentage DPPH scavenging activity of the sample was calculated according to the following equation.

(2) $${DPPH}_{scavenging\;activity}(\%)=(1-\mathrm{As}/\mathrm{Ac})\times 100$$

where As and Ac represent the absorbance of the sample and control, respectively.

### 2.11. ABTS Radical Scavenging Activity

The radical scavenging rate was determined according to Ayyash et al.’s method [30] with modifications. The ABTS working solution was prepared by mixing 7.4 mmol of ABTS and 2.6 mmol of potassium persulfate solution at a ratio of 1:1. Then, the solution was incubated in the dark at room temperature for 12 h. Further, the solution was diluted with ethanol to reach an absorbance of 0.7 at 734 nm. The extraction method used for the supernatant was the same as that described in Section 2.10. A total of 20 μL of the supernatant was mixed with 2 mL of ABTS working solution and incubated in the dark for 6 min. The absorbance was measured at 734 nm and the percentage ABTS scavenging activity of the sample was calculated according to the following equation.

(3) $${ABTS}_{scavenging\;activity}(\%)=(1-\mathrm{As}/\mathrm{Ac})\times 100$$

where As and Ac represent the absorbance of the sample and control, respectively.

### 2.12. Statistical Analysis

All experiments were carried out in triplicate on three separate occasions. Multiple linear regression analysis was used to evaluate the mathematical model of each response [31]. Analysis of variance (ANOVA) and Tukey’s test (*p* < 0.05) were used to evaluate the significance of each response of the model. A polynomial quadratic regression equation (Equation (3)) was used to evaluate the influence of each factor. The R2, adjusted R2, and F-test were used to check the adequacy and significance of the model. The Box–Behnken mixture models and the polynomial quadratic regression equation were analyzed using Design Expert 13 software.

## 3. Results and Discussion

### 3.1. Response Surface Analysis

#### 3.1.1. Model Building and Statistical Significance Test

*L. edodes* (x1), BFSG (x2), DS (x3), and SPI (x4) were taken as factors, and sensory scores were taken as response values. The response surface experiment results are shown in Table 2. Design Expert software was used to perform quadratic regression fitting of the data, and the results are shown in Table 3. The quadratic model was highly significant. The R2 value is 0.9960, which indicates that the experimental value has a good correlation with the predicted response value. The regression of the polynomial model of the sensory evaluation (Y), as expressed by the coding factor (Equation (3)), yielded Y = 9.34 − 0.1517 x1 + 0.0983 x2 + 0.1775 x3 + 0.1075 x4 + 0.0625 x1x2 − 0.0175 x1x3 + 0.020 x1x4 + 0.0475 x2x3 − 0.0450 x2x4 + 0.0175 x3x4 − 0.4333 x1^2^ − 0.1733 x2^2^ − 0.1696 x3^2^ − 0.0571 x4^2^.

#### 3.1.2. Three-Dimensional (3D) Response Surface Plots

**Figure 1:**
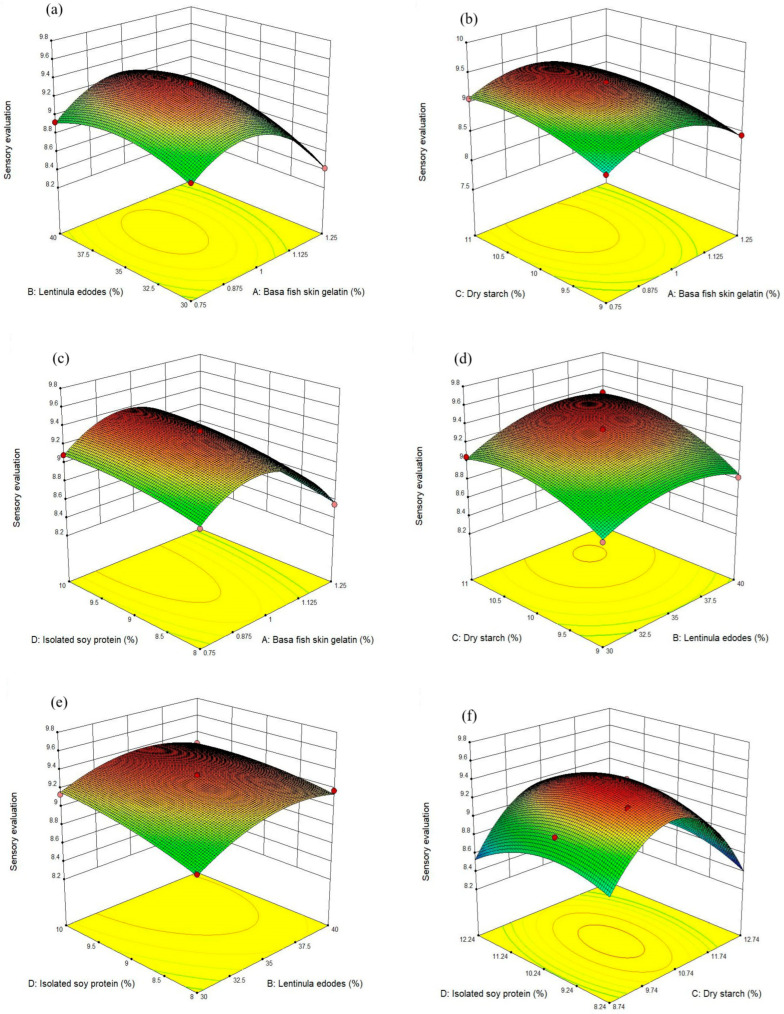
Three-dimensional (3D) response surface plots. (**a**–**f**) is the response surface diagram of the effects of balsa fish skin gelatin and *Lentinula edodes*, balsa fish skin gelatin and dry starch, balsa fish skin gelatin and isolated soy protein, *Lentinula edodes* and dry starch, *Lentinula edodes* and isolated soy protein, dry starch and isolated soy protein on the sensory scores of new sausages, respectively.

**Figure 2:**
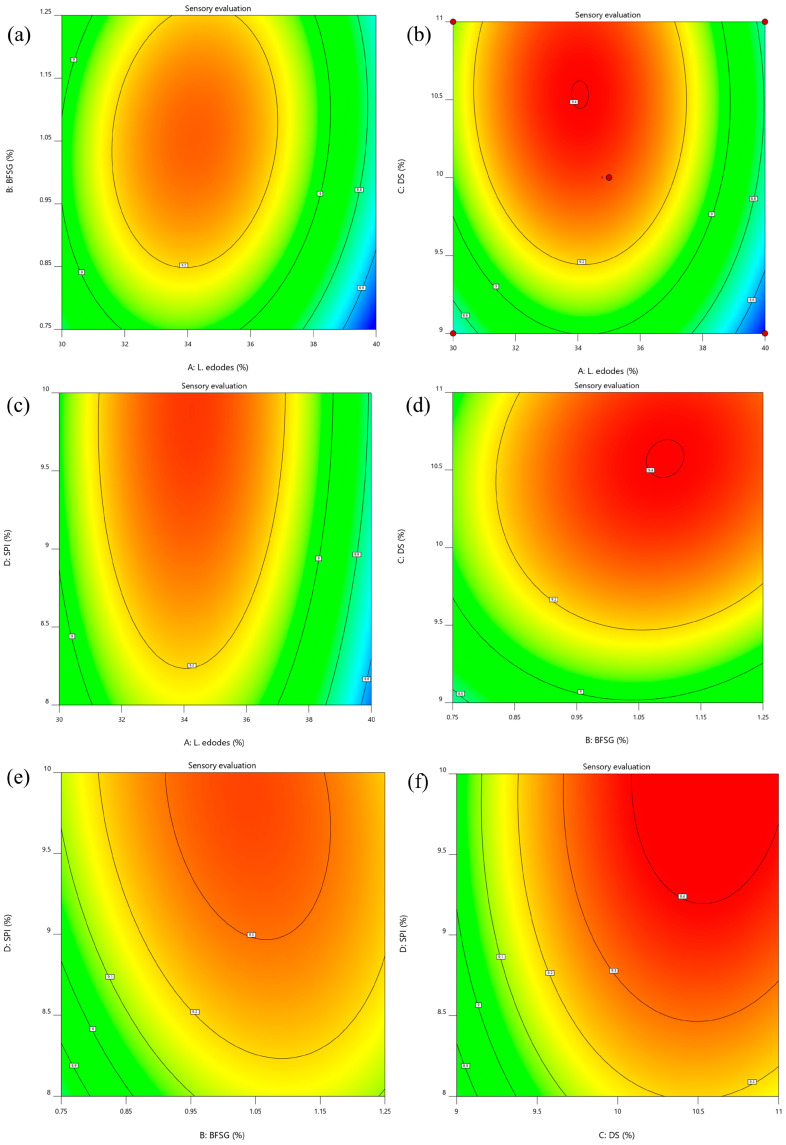
Response surface contour map. (**a**–**f**) is the response surface contour map of the effects of *Lentinula edodes* and balsa fish skin gelatin, *Lentinula edodes* and dry starch, *Lentinula edodes* and isolated soy protein, balsa fish skin gelatin and dry starch, balsa fish skin gelatin and isolated soy protein, dry starch and isolated soy protein on the sensory scores of new sausages, respectively.

The effect of the BFSG and *L. edodes* on the sensory evaluation of samples at a fixed concentration of 10% DS and 9% SPI is presented in Figure 1a and Figure 2a. The maximum sensory evaluation values were observed to be about 1% BFSG and 35% *L. edodes*. The effect of the BFSG and DS on the sensory evaluation of samples at a fixed concentration of 35% *L. edodes* and 9% SPI is presented in Figure 1b and Figure 2b. The maximum sensory evaluation values were observed to be about 1% BFSG and 10% DS. The effect of BFSG and SPI on the sensory evaluation of samples at a fixed concentration of 35% *L. edodes* and 10% DS is presented in Figure 1c and Figure 2c. As the BFSG and SPI concentration increased, the sensory evaluation values of the samples initially increased, followed by a subsequent decrease. These results show that the sensory evaluation reached its highest value at about 1% BFSG and 9% SPI. The effect of *L. edodes* and DS on the sensory evaluation of samples at a fixed concentration of 1% BFSG and 9% SPI is presented in Figure 1d and Figure 2d. The maximum sensory evaluation values were about 35% *L. edodes* and 10% DS. The effect of *L. edodes* and SPI on the sensory evaluation of samples at a fixed concentration of 1% BFSG and 10% DS is presented in Figure 1e and Figure 2e. The maximum sensory evaluation values were about 35% *L. edodes* and 9% SPI. The effect of DS and SPI on the sensory evaluation of samples at a fixed concentration of 35% *L. edodes* and 1% BFSG is presented in Figure 1f and Figure 2f. As the DS and SPI concentration increased, the sensory evaluation values of the samples initially increased, followed by a subsequent decrease. These results show that the sensory evaluation reached its highest value at about 10% DS and 9% SPI.

Overall, considerable interactions exist between each of the independent variables and the sensory evaluation in these 3D response surface plots.

#### 3.1.3. Sensory Evaluation and Validation of the Predicted Optimal Conditions

According to the model equation, the best formulation of the novel sausage was *L. edodes* instead of 36.10% lean meat, 0.96% BFSG, 10.61% DS, and 9.94% SPI. The theoretical sensory evaluation value predicted was 9.47 ± 0.75. To verify the prediction of the model, the optimal preparation conditions of the product were satisfied in three independent replicates. The average sensory evaluation values of the product were 9.51 ± 0.31 (*n* = 3), which is in good agreement with the estimated values of the model equation. These results show that the model in Equation (3) is accurate.

### 3.2. Proximate Composition, Energy Value, and Color

Table 4 shows the proximate composition and energy value of samples. The value of the fat content in the control was 18.33%, which is similar to the finding in our previous study [2], in which we observed a value of 18.49% in the control. Compared with the control, the fat level of the novel sausage was significantly (*p* < 0.05) lower, measuring 3.68% in the sausage (a decrease in the fat content of 79.92%), because the pork fat was replaced by *P. eryngii* in the formulation. According to the literature, the fat content in *P. eryngii* is notably lower than in pork fat [3]. Due to the inclusion of more SPI in the novel sausage, the protein content of the novel sausage was higher than that in the control (16.21% in the sausage, a 28.85% increase). The protein content in the novel sausage was higher than that (15.40 g/100 g) reported by Marti-Quijal et al. [32] and also higher than that in our previous research (9.91 g/100 g) [2]. With regard to water, the novel sausage had a higher value, probably because *L. edodes* and *P. eryngii* have higher water contents than pork fat. This result was lower than beef salami with the fat partially replaced by oyster mushrooms (73.06 g/100 g) [33] and higher than chicken sausages with the chicken meat partially replaced by fresh oyster mushrooms (56.75 g/100 g) [34]. As for the ash content of the samples, there was no significant (*p* > 0.05) difference between the two samples. The results were close to the value of ash content in the Brazilian sausage reported by Souza [35], higher than the value of ash content in the Bologna sausage reported by de Souza Paglarini et al. [36], and lower than the ash content in the low-fat sausage reported by Garcia-Santos, Conceio, Boas, Souza, and Barretto [37].

In addition, the energy content of the control sausage (223.77 kcal/100 g) was close to that of the low-fat Brazilian sausage reported by Souza et al. [36]. The energy of the novel sausage was significantly (*p* < 0.05) different from that of the control group, which was 138.69 kcal per 100 g of sausage (a 38.02% decrease). The energy value of the novel sausage was lower than that of the sausage reported by de Souza Paglarini et al. [38]; specifically, 200–280 kcal/100 g of Bologna sausage in which the fat had been replaced with inulin-based emulsion gels.

Color is one of the most important attributes of meat products. As seen in Table 4, the novel sausage had lower values of L* and a*, but higher b* values than the control in terms of the external color. One possible reason for this was that the lightness of the fried *L. edodes* and *P. eryngii* was lower than that of pork lean meat and fat. Furthermore, the addition of BFSG and more SPI and DS resulted in a decrease in the redness of the novel sausage. The fried *P. eryngii* was yellower than the pork fat, resulting in the novel sausage having more yellowness. Moreover, the internal and external colors of the novel sausages showed the same trend, and the difference between the internal and external colors was attributed to the roasting and smoking of the novel sausage.

### 3.3. Amino Acid Profile

Amino acids play an important role in the nutritional value and sensory evaluation [39]. The results of the amino acid profile (g/100 g) of samples are presented in Table 5. There were no significant differences (*p* < 0.05) between the control and novel sausages in terms of the total, the total essential, and the total non-essential amino acid contents. In the current study, pork lean meat and fat were replaced by *L. edodes* and *P. eryngii* mushrooms and BFSG. So, the amino acids associated with pork lean meat and fat were reduced, while the amino acids associated with the mushrooms and BSFG were increased in the novel sausage. The total amino acid content did not exhibit a significant decrease, indicating that the nutritional value of the novel sausage did not exhibit a noticeable reduction compared with the control group.

Compared with the control, the aspartic acid, glutamic acid, cysteine, and methionine in the novel sausage were increased by 0.38, 0.40, 0.10, and 0.08 g/100 g, respectively, while the levels of valine, alanine, lysine, leucine, histidine, arginine, and tyrosine decreased. This outcome is consistent with our previous studies [2] that replaced pork lean meat with *L. edodes* meat in sausages, increasing the content of most amino acids. This may have been because *L. edodes* and *P. eryngii* mushrooms are rich in those amino acids [2,3]. Except for serine, threonine, and phenylalanine, the other amino acids showed notable differences (*p* < 0.05). In addition, the main amino acids in the optimized novel sausage were glutamic acid, aspartic acid, lysine, leucine, and alanine. The increases in the levels of aspartic acid and glutamic acid are critical for flavor. Aspartic acid and glutamic acid are associated with umami flavor, while alanine, valine, lysine, leucine, histidine, arginine, tyrosine, and cysteine have no direct relationship with flavor. Therefore, the taste of the novel sausages may be better than that of traditional sausages.

### 3.4. Fatty Acid Profile

The fatty acid profile (mg/100 g) of samples is shown in Table 6. The control was abundant in oleic acid, palmitic acid, and linoleic acid. The novel sausage contained high levels of linoleic acid, oleic acid, and palmitic acid. The difference in fatty acids of the two samples suggested that the *L. edodes*, *P. eryngii,* and BFSG, as a substitution for pork lean meat and fat, directly altered the fatty acid profile of the novel sausage. Significant differences (*p* < 0.05) were detected in the saturated fatty acid (SFA), monounsaturated fatty acid (MUFA), and polyunsaturated fatty acid (PUFA) contents between the control and novel sausages. Compared with the control, the SFA of the novel sausage decreased by 1.96%, while the MUFA and PUFA increased by 3.24% and 278.08%, respectively. 

The PUFA/SFA ratio is an important parameter used to determine the nutritional properties of lipids in foods [40]. The Food and Agriculture Organization of the United Nations (FAO) recommends a ratio higher than 0.85 for the human diet. The PUFA/SFA ratio of the novel sausage was 1.88, higher than the control’s 0.49, indicating that the novel sausage has higher nutritional value.

The literature suggests that excessive SFA intake will lead to an increase in the fat content and cholesterol content in the body, which may aggravate the risk of cardiovascular and cerebrovascular diseases [41,42]. Monteiro, Souza, Costa, Faria & Vicente [43] reported that unsaturated fatty acids could help the body decrease the total cholesterol and the plasma low-density lipoprotein (LDL). Several studies have demonstrated the beneficial effect of PUFAs in the prevention of coronary heart diseases by the management of hyperlipidemia and the increase in blood LDL cholesterol. So, the novel sausage may be beneficial to the health of consumers.

### 3.5. Volatile Compound Analysis

Table 7 shows a total of 47 volatile compounds of six groups identified from the two samples, including eight kinds of aldehydes, six kinds of alcohols, eighteen kinds of alkenes, two kinds of ketones, one kind of acid, one kind of ester, and nine kinds of other substances. The main volatile compounds in the control were caryophyllene, copaene, diallyl disulphide, 3-carene, and (E)-cinnamaldehyde, while the main volatile compounds in the novel sausage were alpha-Cubebene, caryophyllene, (E)-cinnamaldehyde, 3-carene, and cadina-3,9-diene. Compared with the control group, the content of hexanal in the novel sausage was significantly increased (*p* < 0.05), while octanal, trans-2-decenal, benzaldehyde, trans-2, trans-4-decadienal, 1-hexanol, and 1-octen-3-ol were all newly detected. This may be because *P. eryngii* completely replaced the pork back fat and *L. edodes* partially replaced the pork lean meat in the sample and these volatile compounds can be found in *P. eryngii* and *P. eryngii* [44,45]. In addition, these volatile compounds may give the novel sausage a new flavor, such as a mushroom and vegetable flavor. The novel sausage had higher levels of aldehydes and ketones, which is in line with the abundance of PUFAs. The alkene content in the optimized sample was higher than that in the control, while the alpha-Cubebene content was the highest. In summary, the content of the optimal aldehydes, alcohols, alkenes, and ketones was improved. Therefore, the increased abundance of aldehydes and ketones in the optimized novel sausage, which can be attributed to the higher content of PUFAs, suggests that the novel sausage may exhibit a richer and more complex flavor characterized by the presence of these volatile compounds. Research indicates that, in the context of preparing stewed goat meat with thyme, octanal, hexanal, and 1-octen-3-ol are crucial components contributing to the distinctive aroma or flavor of the product. These compounds are likely to be considered key elements influencing the sensory perception of stewed goat meat when thyme is utilized as seasoning [46]. The increased levels of these substances in the novel sausage suggest that the flavor of the sausage is enhanced after substitution. At the same time, some volatile compounds in the new type of sausage have also decreased. For example, humulene and copaene, which belong in the “Alcohols” category, were not detected in the novel sausage. These two compounds have grassy, earthy, and woody flavors [47,48,49,50] and may adversely affect the flavor of meat products, resulting in a reduction in the acceptability of meat products. 

### 3.6. Total Phenolic Content (TPC)

The true antioxidant potential is often revealed in terms of the TPC [51]. Table 8 shows the TPC of the samples, which was determined by the regression equation of the calibration curve (y = 0.1497x + 0.1099, R2 = 0.9992). Compared with that of the control, the TPC of the novel sausage was increased by 12.52 times. The TPC value (2.63 mg/g) of the novel sausage was close to that of pork sausage in which the pork lean meat had been replaced by 50% *L. edodes* [2]. Moreover, the TPC in the novel sausage was higher than that in the sausage reported by Fang, Lin, Ha, M, and Warner [52], who incorporated sugarcane fiber into chicken sausage. This is due to the higher content of phenolic compounds in *L. edodes* and *P. eryngii*. The literature suggests that mushrooms usually contain a range of bioactive molecules, such as phenols that provide preventive effects on the degeneration of various human diseases [52]. Based on the findings of the Tawaha study, there is a positive correlation between antioxidant activity and total phenolic content [53]. In summary, the novel sausage, in which *L. edodes* and *P. eryngii* partially replace the pork lean meat and fat, demonstrates excellent antioxidant potential.

### 3.7. DPPH Radical Scavenging Activity

The DPPH free radical scavenging activity is used to evaluate the antioxidant activity [2]. Table 8 shows the DPPH free radical scavenging rate of the samples. Compared with the control, the DPPH free radical scavenging activity of the novel sausage increased by 65.58%. This upward trend is consistent with the TPC and ABTS radical scavenging activity. Wang et al. [2] reported that phenolic compounds are the main source of antioxidant activity. The results in this study were close to those obtained in fish sausages fortified with bee bread extract as prepared by Mohammad, Badrul Hisham, Mustapa, Chan, and Zawawi [4], but higher than the results obtained by Yu, Feng, and Sun [54] in dry fermented sausages, indicating that replacing pork lean meat and fat with *L. edodes* and *P. eryngii* may improve the antioxidant activity of the product, which is conducive to physical health [55].

### 3.8. ABTS Radical Scavenging Activity

Table 8 exhibits the ABTS radical scavenging activity of the samples. Compared with the control, the ABTS free radical scavenging activity of the novel sausage increased by 3.88 times. In addition, it showed a similar trend to those of the TPC and DPPH free radical scavenging activity. This result is in agreement with the findings of Ma et al. [56], who found that the polysaccharide extracted from *P. eryngii* showed ABTS radical scavenging activity. Liu et al. [57] also reported that the polysaccharides from *L. edodes* showed ABTS radical scavenging activity. This may be the reason why the novel sausage had significantly (*p* < 0.05) greater ABTS radical scavenging activity compared with the control [58].

## 4. Conclusions

In the development of a novel type of sausage, pork lean meat was partially substituted with *L. edodes*, while fat was entirely replaced with *P. eryngii* and BFSG. The optimal formulation included 36.10% *L. edodes* as a replacement for lean meat, along with 0.96% balsa fish skin gelatin, 10.61% dry starch, and 9.94% isolated soy protein. A comparative analysis with the control revealed that the novel sausage exhibited reduced fat, energy, and saturated fatty acid levels, but elevated unsaturated fatty acid levels and enhanced antioxidant properties. At the same time, the increase in the ABTS and DPPH indicators in the novel sausage reflects its antioxidant content and the effectiveness of these antioxidants in clearing free radicals. The novel sausage demonstrated higher antioxidant performance and healthier eating characteristics. Overall, these factors demonstrate that the novel sausage is potentially a healthier option for consumers compared with traditional sausages, and the changes in the indicators suggest that the novel sausage may offer a healthier choice for consumers. This study provides new insights into improving the sensory quality of new sausages, promoting the development of healthy sausages in the food industry, and the high-value utilization of edible mushrooms.

## Figures and Tables

**Table 1:** Codes and levels of factors for the response surface design.

Levels			
Variables	−1	0	1
*Lentinula edodes* (*L. edodes*) (%, x1)	30	35	40
Balsa fish skin gelatin (BFSG) (%, x2)	0.75	1	1.25
Dry starch (DS) (%, x3)	9	10	11
Isolated soy protein (SPI) (%, x4)	8	9	10

**Table 2:** Codes and levels of factors for the response surface method experiment.

Run	Independent and Code Variables				Response
	*Lentinula edodes* (*L. edodes*) (%, x1)	Balsa Fish Skin Gelatin (BFSG) (%, x2)	Dry Starch (DS) (%, x3)	Isolated Soy Protein (SPI) (%, x4)	Actual Value
1	0 (35%)	0 (1%)	0 (10%)	0 (9%)	9.35
2	−1 (30%)	0 (1%)	−1 (9%)	0 (9%)	8.7
3	−1 (30%)	0 (1%)	0 (10%)	1 (10%)	9.09
4	0 (35%)	0 (1%)	1 (11%)	−1 (8%)	9.15
5	1 (40%)	0 (1%)	0 (10%)	−1 (8%)	8.55
6	0 (35%)	0 (1%)	1 (11%)	1 (10%)	9.41
7	1 (40%)	0 (1%)	0 (10%)	1 (10%)	8.83
8	0 (35%)	1 (1.25%)	0 (10%)	−1 (8%)	9.18
9	0 (35%)	−1 (0.75%)	−1 (9%)	0 (9%)	8.74
10	−1 (30%)	−1 (0.75%)	0 (10%)	0 (9%)	8.87
11	0 (35%)	0 (1%)	−1 (9%)	1 (10%)	9.05
12	0 (35%)	−1 (0.75%)	1 (11%)	0 (9%)	9.05
13	0 (35%)	0 (1%)	0 (10%)	0 (9%)	9.33
14	0 (35%)	0 (1%)	−1 (9%)	−1 (8%)	8.86
15	−1 (30%)	0 (1%)	1 (11%)	0 (9%)	9.07
16	0 (35%)	−1 (0.75%)	0 (10%)	1 (10%)	9.14
17	0 (35%)	1 (1.25%)	−1 (9%)	0 (9%)	8.83
18	1 (40%)	0 (1%)	−1 (9%)	0 (9%)	8.45
19	0 (35%)	1 (1.25%)	0 (10%)	1 (10%)	9.27
20	0 (35%)	−1 (0.75%)	0 (10%)	−1 (8%)	8.87
21	1 (40%)	−1 (0.75%)	0 (10%)	0 (9%)	8.42
22	−1 (30%)	0 (1%)	0 (10%)	−1 (8%)	8.89
23	1 (40%)	1 (1.25%)	0 (10%)	0 (9%)	8.73
24	0 (35%)	1 (1.25%)	1 (11%)	0 (9%)	9.33
25	0 (35%)	0 (1%)	0 (10%)	0 (9%)	9.34
26	1 (40%)	0 (1%)	1 (11%)	0 (9%)	8.75
27	−1 (30%)	1 (1.25%)	0 (10%)	0 (9%)	8.93

**Table 3:** Analysis of variance (ANOVA) of items in the regression equation.

Source of Variation	Sum of Squares	Degree of Freedom	Mean Square	F-Value	*p*-Value	Significance
Model	2.02	14	0.1442	199.05	<0.0001	Significant
x1	0.2760	1	0.2760	381.10	<0.0001	**
x2	0.1160	1	0.1160	160.20	<0.0001	**
x3	0.3781	1	0.3781	521.98	<0.0001	**
x4	0.1387	1	0.1387	191.46	<0.0001	**
x1x2	0.0156	1	0.0156	21.57	0.0006	**
x1x3	0.0012	1	0.0012	1.69	0.2179	
x1x4	0.0016	1	0.0016	2.21	0.1630	
x2x3	0.0090	1	0.0090	12.46	0.0041	**
x2x4	0.0081	1	0.0081	11.18	0.0058	**
x3x4	0.0012	1	0.0012	1.69	0.2179	
x1^2^	1.00	1	1.00	1382.68	<0.0001	**
x2^2^	0.16	1	0.16	221.23	<0.0001	**
x3^2^	0.15	1	0.15	211.76	<0.0001	**
x4^2^	0.02	1	0.02	23.99	0.0004	**
Residual	0.0087	12	0.0007			
Lack of fir	0.0085	10	0.0008	8.49	0.1099	Not significant
Sum	2.03	26				
			R2 = 0.9957		R2Adj = 0.9907	

Values are given as the mean ± standard error. **: *p* < 0.01.

**Table 4:** Proximate composition (%), energy value (kcal/100 g), and color parameters of the novel sausage.

Parameters	Control	Novel Sausage
Fat	18.33 ± 0.19 ^b^	3.68 ± 0.08 ^a^
Protein	12.58 ± 0.29 ^a^	16.21 ± 0.25 ^b^
Water	63.95 ± 0.23 ^a^	66.91 ± 0.84 ^b^
Ash	3.02 ± 0.06 ^a^	2.99 ± 0.06 ^a^
Energy value	223.77 ± 0.26 ^b^	138.69 ± 0.18 ^a^
External color		
L*	46.03 ± 0.18 ^b^	44.32 ± 0.21 ^a^
a*	18.45 ± 0.20 ^b^	18.02 ± 0.15 ^a^
b*	18.72 ± 0.09 ^a^	19.20 ± 0.12 ^b^
Internal color		
L*	55.32 ± 0.13 ^b^	53.25 ± 0.16 ^a^
a*	16.64 ± 0.06 ^b^	16.12 ± 0.10 ^a^
b*	14.28 ± 0.05 ^a^	14.73 ± 0.08 ^b^

Values are given as the mean ± standard error. a, b: different letters in the same row indicate statistical differences (*p* < 0.05).

**Table 5:** Amino acid profile (g/100 g) of the novel sausage.

Parameters	Control	Novel Sausage
Essential		
Valine	0.89 ± 0.05 ^b^	0.84 ± 0.00 ^a^
Methionine	0.06 ± 0.00 ^a^	0.14 ± 0.00 ^b^
Isoleucine	0.74 ± 0.05 ^b^	0.72 ± 0.03 ^a^
Phenylalanine	0.84 ± 0.04 ^a^	0.83 ± 0.02 ^a^
Lysine	1.29 ± 0.09 ^b^	1.02 ± 0.01 ^a^
Leucine	1.12 ± 0.08 ^b^	1.09 ± 0.01 ^a^
Threonine	0.73 ± 0.05 ^a^	0.73 ± 0.01 ^a^
Total EAAs	5.67 ± 0.36 ^a^	5.37 ± 0.08 ^a^
Nonessential		
Aspartic	2.04 ± 0.16 ^a^	2.42 ± 0.02 ^b^
Glutamic	3.53 ± 0.25 ^a^	3.93 ± 0.03 ^b^
Serine	0.85 ± 0.04 ^a^	0.85 ± 0.02 ^a^
Histidine	0.84 ± 0.02 ^b^	0.75 ± 0.03 ^a^
Glycine	1.01 ± 0.02 ^b^	0.98 ± 0.00 ^a^
Arginine	0.81 ± 0.04 ^b^	0.72 ± 0.02 ^a^
Alanine	1.11 ± 0.06 ^b^	1.03 ± 0.01 ^a^
Tyrosine	0.39 ± 0.03 ^b^	0.22 ± 0.02 ^a^
Cysteine	0.03 ± 0.01 ^a^	0.13 ± 0.01 ^b^
∑NEAA	10.61 ± 0.63 ^a^	11.03 ± 0.16 ^a^
Total FAA	16.28 ± 0.99 ^a^	16.4 ± 0.24 ^a^

Values are given as the mean ± standard error. a, b: different letters in the same row indicate significant differences (*p* < 0.05).

**Table 6:** Fatty acid profile (mg/100 g) of the novel sausage.

Parameters	Control	Novel Sausage
C14:0	2.71 ± 0.01 ^a^	1.56 ± 0.07 ^b^
C15:0	ND	0.19 ± 0.01
C16:0	45.46 ± 0.69 ^b^	48.17 ± 0.23 ^a^
C17:0	0.41 ± 0.04 ^a^	0.21 ± 0.03 ^b^
C18:0	19.87 ± 0.09 ^a^	16.67 ± 0.09 ^b^
C20:0	ND	0.31 ± 0.02
C16:1 n5	0.34 ± 0.04 ^a^	0.26 ± 0.04 ^a^
C16:1 n7	4.55 ± 0.13 ^a^	3.19 ± 0.07 ^b^
C18:1	76.69 ± 0.72 ^b^	82.10 ± 0.70 ^a^
C20:1	1.97 ± 0.01 ^a^	0.71 ± 0.05 ^b^
C18:2	32.83 ± 0.18 ^b^	121.70 ± 0.67 ^a^
C20:4	0.52 ± 0.02 ^b^	4.39 ± 0.13 ^a^
SFA	68.45 ± 0.83 ^a^	67.11 ± 0.45 ^b^
MUFA	83.55 ± 0.9 ^b^	86.26 ± 0.86 ^a^
PUFA	33.35 ± 0.20 ^b^	126.09 ± 0.80 ^a^
PUFA/SFA	0.49	1.88

Values are given as the mean ± standard error. a, b: different letters in the same row indicate statistical differences (*p* < 0.05). SFA, saturated fatty acid; MUFA, monounsaturated fatty acid; PUFA, polyunsaturated fatty acid.

**Table 7:** Volatile compounds (%) of the novel sausage.

NO.	Compound	Control	Novel Sausage
	Aldehydes		
1	Hexanal	1.55 ± 0.09 ^a^	2.22 ± 0.13 ^b^
2	Decanal	1.01 ± 0.06 ^b^	0.67 ± 0.05 ^a^
3	Nonanal	2.01 ± 0.05 ^b^	1.82 ± 0.10 ^a^
4	Octanal	ND	0.87 ± 0.02
5	trans-2-Decenal	ND	0.34 ± 0.01
6	Benzaldehyde	ND	1.12 ± 0.06
7	trans-2, trans-4-Decadienal	ND	0.69 ± 0.06
8	(E)-Cinnamaldehyde	8.97 ± 0.35 ^b^	7.47 ± 0.15 ^a^
	Total	13.53	15.21
	Alcohols		
9	Syringol	0.87 ± 0.21 ^a^	0.98 ± 0.06 ^b^
10	Cinnamyl alcohol	3.60 ± 0.33 ^b^	3.16 ± 0.54 ^a^
11	Estragole	0.41 ± 0.06 ^a^	0.50 ± 0.02 ^b^
12	Linalool	2.02 ± 0.18 ^b^	1.69 ± 0.17 ^a^
13	1-Hexanol	ND	0.51 ± 0.05
14	1-Octen-3-ol	ND	0.72 ± 0.06
	Total	6.90	7.55
	Alkenes		
15	3-Carene	9.78 ± 0.67 ^b^	5.53 ± 0.76 ^a^
16	Muurolene	1.62 ± 0.28 ^a^	2.45 ± 0.32 ^b^
17	Cadinene	0.70 ± 0.03 ^a^	1.11 ± 0.16 ^b^
18	Selinene	0.55 ± 0.08 ^b^	0.36 ± 0.03 ^a^
19	Guaiene	1.23 ± 0.19 ^a^	1.38 ± 0.14 ^b^
20	Caryophyllene	20.92 ± 1.32 ^b^	19.34 ± 1.76 ^a^
21	alpha-Phellandrene	2.16 ± 0.85	ND
22	Ocimene	1.04 ± 0.11	ND
23	Cadina-3,9-diene	2.09 ± 0.78 ^a^	4.22 ± 0.98 ^b^
24	1R-.alpha.-Pinene	0.86 ± 0.02	ND
25	Copaene	14.36 ± 1.71	ND
26	Humulene	1.38 ± 0.14	ND
27	Terpinen	ND	3.29 ± 1.12
28	Phellandrene	ND	1.00 ± 0.09
29	(+)-Cyclosativene	ND	0.54 ± 0.02
30	alpha-Cubebene	ND	22.04 ± 1.65
31	Eudesmene	ND	0.60 ± 0.04
32	Cubebene	ND	0.78 ± 0.03
33	Isoterpinolene	ND	0.90 ± 0.06
	Total	56.68	63.52
	Ketones		
34	3,4-Dimethylacetophenone	ND	0.53 ± 0.03
35	Phloretin	0.54 ± 0.03 ^a^	0.61 ± 0.02 ^b^
	Total	0.54	1.14
	Ester		
36	Hexadecanoic acid, methyl ester	0.45 ± 0.06	ND
	Total	0.45	ND
	Acids		
37	4-Hydroxyaminobenzoic acid	0.70 ± 0.06	ND
	Total	0.45	ND
	Others		
38	Anethole	1.86 ± 0.23 ^a^	2.74 ± 0.25 ^b^
39	Diallyl disulphide	12.58 ± 0.73 ^a^	3.98 ± 0.94 ^b^
40	o-Cymene	0.95 ± 0.06 ^a^	1.15 ± 0.23 ^b^
41	4-Methoxy-3-(methoxymethyl)phenol	0.84 ± 0.04 ^a^	0.98 ± 0.06 ^b^
42	1,3-Dithiane	0.84 ± 0.05	ND
43	Bicyclo [3.1.1]heptane, 6,6-dimethyl-2-methylene-, (1S)-	2.21 ± 0.87	ND
44	Cajeputen	1.26 ± 0.27 ^a^	2.55 ± 0.04 ^b^
45	2-Amylfuran	ND	0.85 ± 0.03
46	4-Carvomenthenol	ND	0.34 ± 0.01
47	m-Cymene	0.66 ± 0.01	ND

Values are given as the mean ± standard error. a, b: different letters in the same row indicate statistical differences (*p* < 0.05). ND, not detected.

**Table 8:** Total phenolic content (TPC), DPPH scavenging activity, and ABTS scavenging activity of the novel sausage.

Parameters	Control	Novel Sausage
Total phenolic content (TPC) (mg/g)	0.21 ± 0.01 ^a^	2.63 ± 0.08 ^b^
DPPH radical scavenging activity (%)	48.55 ± 0.17 ^a^	80.39 ± 0.76 ^b^
ABTS radical scavenging activity (%)	5.35 ± 0.11 ^a^	26.29 ± 0.25 ^b^

Values are given as the mean ± standard error. a, b: different letters in the same row indicate statistical differences (*p* < 0.05).

## Data Availability

Data is contained within the article.
